# Clinical Management of Dens Invaginatus Type 3: A Case Report

**Published:** 2011-08-15

**Authors:** Elham Shadmehr, Ali Reza Farhad

**Affiliations:** 1. Department of Endodontics, School of Dentistry, Isfahan University of Medical Sciences, Isfahan, Iran.; 2. Department of Endodontics, Dental Research Center, School of Dentistry, Isfahan University of Medical Sciences, Isfahan, Iran.

**Keywords:** Canine Teeth, Dens in Dente, Invagination

## Abstract

Dens invagination (DI) is a developmental abnormality of teeth which frequently results in a complex internal anatomy of the root canal system. DI type 3 is an anomaly characterized by infolding of enamel and dentin extending into the root apex. This may present difficulties when forming a diagnosis and treatment plan. Many treatment modalities have been presented in case reports for DI type 3, but there is insufficient evidence to recommend a therapy. This case report presents the successful nonsurgical root canal treatment of a maxillary canine with an open apex DI type 3, necrotic pulp, and an associated large periradicular lesion.

## INTRODUCTION

Dens invagination (DI) or “dens in dente” is one of the rare developmental abnormalities of teeth with a wide range of morphological variations. Salter described it as “a tooth within a tooth” for the first time [1]. The most acceptable etiologic theory is that DI results from an infolding of the enamel organ (outer portion) into the dental papilla (inner portion) during tooth development with the formation of a pocket [2]. Other etiologic factors include incomplete lateral fusion of two germs, abnormal pressure from the surrounding tissues during dental development, the distortion of the enamel organ during tooth formation, the constriction of the dental arch in the enamel organ, and a retardation or acceleration of growth of the internal enamel epithelium [3][4][5][6][7].

DI may occur in any deciduous or permanent teeth [8]. It has a varied prevalence which ranges between 0.04-10% [9]. The maxillary lateral incisor is most commonly affected, followed by central incisors and less frequently the canines [10]. Bilateral appearance is common in maxillary lateral incisors [11].

Oehlers classified DI into three types according to the depth of the invagination into the root [4]. In type 1, the invagination is confined within the crown and does not extend beyond the CEJ; in type 2, invagination invades the root as a blind sac, with or without connection to the dental pulp; and finally in type 3, invagination penetrates through the root and forms a second foramen in the apex or along the root, in the periodontium.

Teeth with DI are prone to early caries and pulp necrosis. The treatment plan for necrotic pulp cases can be conservative root canal therapy, endodontic surgery, intentional replantation, or extraction of the affected teeth. Generally DI, especially DI type 3 is treated surgically; however, it is possible to perform conservative nonsurgical root canal treatment (NSRCT) successfully. Due to irregular anatomy and limited access, complete debridement of the. root canal system seems impossible. To overcome these limitations, applying intracanal medicament might be useful. The following case report presents the clinical management of a rare DI type 3 in a maxillary canine with an open apex, necrotic pulp and associated large periradicular lesion.

## CASE REPORT

**Figure 1:**
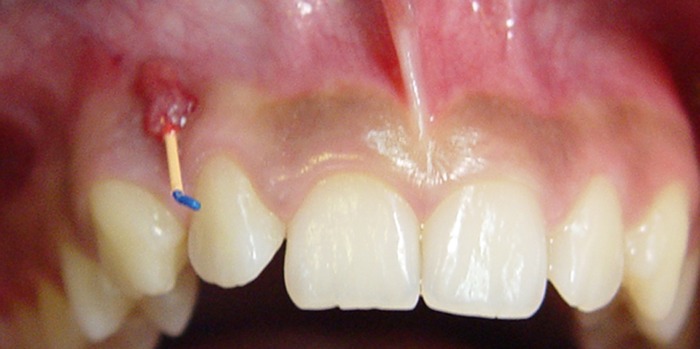
Hyperplastic tissue covering the sinus tract which was traced with a gutta-percha point.

**Figure 2:**
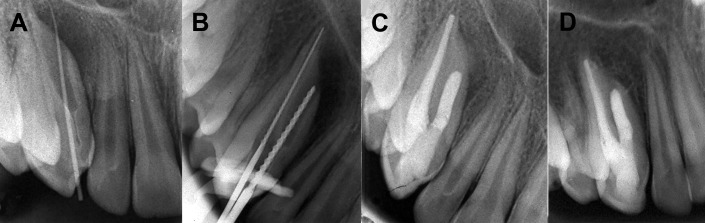
A: Periapical radiograph showing a gutta-percha point tracing the sinus tract and enamel like tract which is extended toward the invaginated portion. B: Length determination of the main and invaginated canals. C: Immediate postoperative periapical radiograph taken after final canal obturation and placement of coronal seal. D: 18 months follow-up radiograph demonstrating complete periapical healing.

A healthy 12-yr-old male patient with a history of a sinus tract in the upper right anterior mucosa was referred to the author’s private clinic. Medical history was unremarkable. Intra-oral soft tissues were free of any pathologic signs and there was no associated swelling. Clinical examination revealed a sinus tract in the buccal mucosa next to teeth #6 and #7. A gutta-percha point (Dentsply, Maillefer, OK, USA) was inserted into the sinus tract and a radiograph was taken to trace the gutta-percha point (Figure 1). The origin of the sinus tract was located apical to tooth #6. The radiograph showed a mature canine with an open apex DI (type 3 Oehlers). An extended area of radio-lucency adjacent to the mesial aspect of the apex was noted. The radiograph also demonstrated an enamel-like root canal tract that appeared to be separate from the main root canal system (two canals) (Figure 2A).

The tooth responded to thermal and electrical tests, and periodontal probing revealed normal periodontium. There was no mobility, no pain on palpation, and tenderness to percussion was mild. The crown of the affected tooth was intact; however it displayed a lingual pit above the cingulum. The affected canine was somewhat larger in its buccolingual aspect than its counterpart. The main canal of the pulp was diagnosed as normal and the accessory root canal tract was diagnosed as having a necrotic pulp associated with DI type 3 and chronic apical periodontitis.

NSRCT was planned initially with possible surgical intervention in the future. Both parents and patient were subsequently informed about the complex anatomy of the tooth and the long-term prognosis of different treatment plans, and a decision was made to perform nonsurgical RCT. A written consent form was acquired before each phase of the treatment. The tooth was anesthetized with 2% lidocaine with 1:100,000 epinephrine (Xylocaine, Dentsply Pharmaceutical, York, PA, USA) and isolated with a rubber dam. Access cavity was made from the lingual pit with high speed turbine and diamond fissure bur (Dentsply, Maillefer, Baillaigues, Switzerland). Despite all efforts to limit the access to the invaginated portion, the main root canal was exposed and the presence of vital pulp tissue and bleeding confirmed the initial diagnosis. Subsequently, DI was exposed which resulted in drainage from this infected canal. Working lengths for both canals were determined radiographically (Figure 2B). Gates Glidden drills and hand k-files (Dentsply, Maillefer, USA) were used to clean and shape canals. Irrigation was performed with copious 0.5% NaOCl solution. Calcium hydroxide (Pulpdent Corp, Watertown, MA, USA) was placed in the main canal and invagination for one week. The access cavity was sealed with Cavit (3M, ESPE, Seefeld, Germany) between appoint-ments. At the next visit, the sinus tract had healed. However, there was still some serous discharge and bleeding through the invaginated canal. After irrigation with 0.5% NaOCl, calcium hydroxide was replaced in both canals for further one week.

At the third appointment, intracanal medicament was removed, and both canals were made completely dry. Due to the blunderbuss nature of the invaginated portion, mineral trioxide aggregate (MTA) was used to obturate this canal. The invaginated and main canals were dried with paper points (Dentsply, Maillefer, OK, USA). First a calcium hydroxide plug was placed in the invaginated canal to prevent extrusion of MTA. Then, ProRoot MTA (Maillefer, Dentsply, Baillaigues, Switzerland) was mixed with normal saline according to manufacturer’s instruction and was used to obturate the entire invaginated canal. Main root canal with the closed apex was obturated with gutta-percha (Dentsply, Maillefer,OK, USA) and AH-26 sealer (Dentsply, DeTrey, Konstanz, Germany) by lateral condensation technique. The access cavity was restored with composite resin (Solitaire 2, Heraeus Kulzer, Wehrheim, Germany) (Figure 2C).

The 18 months follow-up clinical examination revealed healthy clinical appearance and function; radiographs showed complete healing of the periapical pathology (Figure 2D).

## DISCUSSION

In this case of DI, root development of invaginated portion was disrupted by pulp necrosis due to a deep pit between the invaginated cavity and the pulp chamber. Once invaginated teeth erupt into the oral cavity, the irregularities act as a haven for microorganism colonization thereby causing pulp necrosis, subsequent periapical involvement and possible retardation of root development [12].

In DI cases in which necrosis is established different methods of therapy (conservative root canal therapy, endodontic surgery, intentional replantation, or extraction) can be considered. In such cases, conservative NSRCT is difficult because of the unpredictable shape of the internal anatomy [13]. Despite the difficulties in cleaning and shaping of the unusual internal anatomy of the present case and the large periradicular lesion, RCT was attempted. This decision was based on the many case reports demonstrating success with this treatment [14]. Moreover, NSRCT was selected over other treatment modalities due to the patient’s age, and because surgical interventions can be used later if conservative treatment failed.

Interestingly, the success of this case indicates that the size of the periradicular lesion does not dictate the treatment procedure or influence the treatment outcome of NSRCT.

In the present case, to compensate for the shortcomings of canal preparation inter-appointment medicament was considered. Calcium hydroxide was used for its anti-microbial action, tissue dissolving effect and to control exudation from the invaginated canal [13]. Furthermore, calcium hydroxide has favorable effects on the healing of periradicular lesion by increasing the pH of the periapical environment and by providing calcium ion for the repair process [15]. Also, calcium hydroxide has denaturating effect on proinflammatory mediators such as IL1 and TNF [16].

Another challenge for this case was the open apex of the invaginated portion. Incompletely formed roots need modified obturation pro-cedures due to thin dentin walls and difficulties in controlling the apical extrusion of the filling material. Different methods of treatment can be considered in open apex teeth with necrotic pulp such as apexification, MTA plug and revascularization.

Apexification with calcium hydroxide has been shown to encourage cementogenesis and osteogenesis [17][18]. Ferguson et al. and Schindler et al. used calcium hydroxide in teeth with dens tracts for apexification [19][20]. The creation of hard tissue barrier using calcium hydroxide, although quite predictable, takes about 3 to 18 months during which there is a risk of tooth fracture [21]. The use of MTA as an alternative treatment for immature roots has been reported [22][23]. MTA has been shown to consistently induce apical hard tissue formation due to its good sealing ability and high biocompatibility [24]. In this case, the total length of the invaginated portion was obturated with MTA as retention was not required for restorative purposes; moreover, greater thickness of MTA provides a more superior seal.

Recently, revascularization procedures have been introduced for the treatment of necrotic teeth with open apex [25][26]. Since DI cases present a more complex internal anatomy, disinfection of the root canal system can be a challenge. For tissue regeneration a properly disinfected root canal system is essential, and therefore the invaginated portion of this tooth might not have been a suitable candidate for regenerative endodontics.

## CONCLUSION

This case illustrates that even in teeth with open apex DI type 3 and associated large periradicular lesion, a proper NSRCT can result in a successful outcome.
